# Atlanta Medical College

**Published:** 1868-02

**Authors:** 


### EDITORIAL AND MISCELLANEOUS.

### ATLANTA MEDICAL COLLEGE.

From the opening of this institution in May 1855, the facilities now offered have not been equalled. All the advantages heretofore possessed, with the additional convenience of a preparatory course, continuous from the close of the regular session to the commencement of the next, are now presented to the student of Medicine.

The operations of the preparatory term, which includes about eight months, have been thoroughly tested the present winter, and prove to be all the learner requires in a course of preparatory study. Not only so, but the daily opportunity of witnessing the treatment of ten to twenty cases presented in the Dispensary, and about one hundred in the Hospital, gives unusual advantages in the study of Practical or Clinical Medicine, to those more advanced. This arrangement for clinical study continues throughout the year, and provision is now made for it in the curriculum of studies during the regular course, the Chair of which will be filled by the experienced and popular teacher, Prof. H. V. M. Miller. This branch, has not, heretofore, been taught in the form of regular didactic course, but will have devoted to it during the next regular session, equal attention with that of any other department of the College.

After the Annual Announcement of Lectures for 1868 was sent to press, several changes occurred in the Faculty. As above indicated Professor Miller has consented to discharge the laborous and responsible duties connected with the chair of Clinical Medicine. Prof. Miller's extensive medical information, and ample experience as a teacher in Augusta and Memphis, and as a civil and military practitioner of Medicine and Surgery, admirably adapts him to the position.

Prof. Jesse Boring accepts the chair of Obstetrics and Diseases of Women and Children, the position he now occupies in the new Medical College, founded by himself, if we are not mistaken, in Galveston, Texas. We know of Prof. Boring as a teacher of Obstetrics. Those who attended the first and second sessions of the Atlanta Medical College, in 1855 and 1856 can bear testimony to the attractive eloquence and thoroughness of instruction which characterized his lectures during the two courses referred to. He will again make his home in Atlanta, and be ready to enter upon his duties in the College next May.

Dr. John M. Johnson accepts the chair of Physiology, in place of Dr. Hillyer, resigned. Dr. Johnson, a refugee from his native home in Kentucky, at the commencement of the war, has been made familiar to many in the South, as Surgeon in various parts of the Army of Tennessee, and at the Post of Atlanta, and now as one of the Editors of this Journal.

The corps of Professors thus organized will bring into the duties of their respective chairs all the energy necessary to thorough instruction.

